# Microarray Analysis on Gene Regulation by Estrogen, Progesterone and Tamoxifen in Human Endometrial Stromal Cells

**DOI:** 10.3390/ijms16035864

**Published:** 2015-03-13

**Authors:** Chun-E Ren, Xueqiong Zhu, Jinping Li, Christian Lyle, Sean Dowdy, Karl C. Podratz, David Byck, Hai-Bin Chen, Shi-Wen Jiang

**Affiliations:** 1 Department of Obstetrics and Gynecology, Center of Reproductive Medicine, Affiliated Hospital of Weifang Medical University, Weifang 261043, China; E-Mail: ren@wfmc.edu.cn; 2 Department of Obstetrics and Gynecology, the Second Affiliated Hospital of Wenzhou Medical University, Wenzhou 325001, China; E-Mail: wzzxq@wzhealth.com; 3 Department of Biomedical Science, Mercer University School of Medicine, Savannah, GA 31404, USA; E-Mail: li_j@mercer.edu or li.jinping@mayo.edu; 4 Department of Obstetrics and Gynecology, Memorial Health University Medical Center, Savannah, GA 31404, USA; E-Mail: davidbyck@memorialhealth.com; 5 Department of Biochemistry and Molecular Biology, Mayo Clinic, Rochester, MN 55905, USA; 6 Department of Biology, Savannah State University, Savannah, GA 31419, USA; E-Mail: clyle0408@gmail.com; 7 Department of Obstetrics and Gynecology, Mayo Clinic, Rochester, MN 55905, USA; E-Mails: dowdy.sean@mayo.edu (S.D.); podratz.karl@mayo.edu (K.C.P.); 8 Department of Histology and Embryology, Shantou University Medical College, Shantou 515041, China; 9 Curtis and Elizabeth Anderson Cancer Institute, Department of Laboratory Oncology Research, Memorial University Medical Center, Savannah, GA 31404, USA

**Keywords:** stroma, uterus, estrogen, progesterone, tamoxifen, transcription

## Abstract

Epithelial stromal cells represent a major cellular component of human uterine endometrium that is subject to tight hormonal regulation. Through cell-cell contacts and/or paracrine mechanisms, stromal cells play a significant role in the malignant transformation of epithelial cells. We isolated stromal cells from normal human endometrium and investigated the morphological and transcriptional changes induced by estrogen, progesterone and tamoxifen. We demonstrated that stromal cells express appreciable levels of estrogen and progesterone receptors and undergo different morphological changes upon hormonal stimulation. Microarray analysis indicated that both estrogen and progesterone induced dramatic alterations in a variety of genes associated with cell structure, transcription, cell cycle, and signaling. However, divergent patterns of changes, and in some genes opposite effects, were observed for the two hormones. A large number of genes are identified as novel targets for hormonal regulation. These hormone-responsive genes may be involved in normal uterine function and the development of endometrial malignancies.

## 1. Introduction

In human endometrium the epithelial glandular cells are surrounded by a rich mesenchymal component that contains fibroblast-like stromal cells. Morphological changes in both epithelial and stromal cells during menstrual cycles have been well documented [1,2,3]. In the proliferative phase, estrogen exerts mitogenic effects on both cell groups, leading to a quick expansion of glandular epithelial and stromal cells [4]. The increased levels of progesterone in the secretory phase suppress cell growth but promote differentiation [5]. At the end of the secretory phase, endometrial gland maturation is accompanied by predecidualization changes of stromal cells around blood vessels. During the decidualization process stromal cells become terminally differentiated upon implantation. In the absence of implantation, withdrawal of ovarian hormones leads to shedding of the endometrium, which marks the completion of a menstrual cycle [6,7]. Thus, steroid hormones serve as a driving force behind the synchronized actions of stromal and epithelial glandular cells. However, most previous studies concentrated on epithelial cells with relatively little attention paid to the hormonal response by stromal cells. The molecular events controlled by ovarian hormones in stromal cells, such as those occurring on the gene transcription level, remain largely unknown.

In addition to their function of supporting normal reproductivity, stromal cells and their responses to ovarian steroids are involved in the pathogenesis of endometrial hyperplasia and endometriosis. It has been observed that synthetic progestins such as levonorgestrel and oral medroxyprogesterone acetate significantly affect the expression of estrogen and progesterone receptors in glandular and stromal cells [8]. This indicates the presence of autoregulatory feedback loops that may amplify hormonal responses in these cells. Interestingly, endometrial stromal cells isolated from women with endometriosis exhibited aberrant responses to ovarian hormones in the migratory and invasive behaviors, suggesting the involvement of these cells in the pathogenesis of endometriosis that is characterized by ectopic endometrial overgrowth [9].

Endometrial stromal sarcoma is a rare but life-threatening uterine malignancy with a poor prognosis [10]. Current radiation and chemotherapy regimens are relatively ineffective for treating stromal sarcomas [11]. Malignant mixed mullerian tumor, also referred to as carcinosarcoma, represents a highly aggressive uterine malignancy containing both carcinomatous and sarcomatous components [12]. While most carcinosarcomas are believed to arise from monoclonal endometrial stem cells, some cases may be true collision tumors derived from epithelial and stromal cells independently [12]. It is noteworthy that elevated estrogen levels associated with obesity and nulliparity represent the most recognized risk factor for stromal sarcoma and mixed mullerian tumors [13]. Exposure to exogenous estrogenic compounds, including the use of oral contraceptives, postmenopausal hormone replacement therapy [14], and use of tamoxifen for breast cancer treatment [15], have all been linked to an increased risk for stromal sarcomas. These observations underscore the significance of carcinogenic effects by estrogens in human endometrial stromal cells.

An increasing body of evidence supports the involvement of mesenchymal-epithelial interactions in cancer development [16]. In prostate cancers, steroid-induced production of EGF and TGF-α by stromal cells is considered to be a major factor for continued cancer expansion [17]. This paracrine action may contribute to a seemingly androgen-independent growth of epithelial cells, and account for the failure of predicting the response to endocrine therapies merely based on the androgen receptor levels of cancer cells [17]. More interestingly, frequent loss of heterozygosity (LOH) has been found in normal-appearing stromal cells micro-dissected from mammary ductal carcinomas. This finding suggests that abnormal stromal-epithelial interactions may play a significant part in the progression of mammary neoplasia, and alterations in stromal cells may precede the epithelial transformation [18]. *In vitro* studies have shown that uterine stromal cells are able to promote the growth [19] and invasiveness [20] of endometrial cancer cells when they were co-cultured in a three-dimensional model. In this system, matrix metalloproteinases (MMPs) produced by normal stromal cells were found to translocate to the surface of endometrial adenocarcinoma cells. Interestingly, MMP-2 secretion and translocation in the co-cultures were greatly enhanced by estrogen [20]. It was also reported that stromal cells produce vascular endothelial growth factor and soluble vascular endothelial growth factor receptor, and the expression of these factors are regulated by estrogen and selective estrogen receptor modulators [21]. Taken together, these findings suggested that endometrial stromal cells represent an important regulation target of ovarian hormones, and aberrant hormonal regulation may contribute to the development of endometrial malignancies.

In this study, we used primary stromal cell culture as a study model to investigate the morphological as well as transcriptome changes induced by estrogen, progesterone, and tamoxifen. Changes of gene expression in response to these hormones were detected, validated, and compared. Numerous genes governing diversified cell functions were found to be targets for hormonal regulation. A large number of novel genes with unknown functions were also identified. These findings provided useful information for studying the pathophysiological functions of stromal cells in the normal endometrium and the development of uterine hyperplasia and neoplasia.

## 2. Results

### 2.1. Expression of Estrogen and Progesterone Receptors in Stromal Cell Cultures

**Figure 1:**
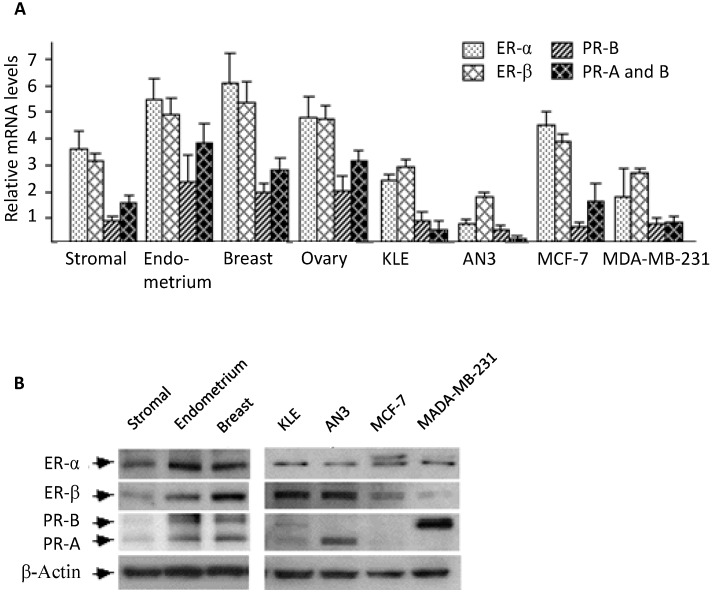
Expression of estrogen and progesterone receptors in human stromal cells. (**A**) Results of real-time PCR. ER-α, ER-β, total PR, and PR-A mRNA levels were measured in stromal cell primary culture, whole tissues from endometrium, breast, ovary, and cancer cells lines KLE, AN3, MCF-7, and MDA-MB-231. Average levels and standard errors were presented; (**B**) Western blotting analysis of ER and PR protein expression in stromal cell culture. Western blotting was performed with specific antibodies raised against ER-α, ER-β, and PR (detecting both **A** and **B** isoforms).

Stromal cell populations represent a large majority of cells in human endometrium. The protocol for stromal cell isolation has long been established and successfully used by many laboratories. Consistent with previous reports, we observed that the isolated stromal cells displayed fibroblastic morphology and a high homogeneity, indicating a high purity of the population. To collect background information we measured ER and PR expression levels in stromal cultures. Real-time PCR results (Figure 1A) indicated that when compared to breast, ovary, and whole endometrium tissues, stromal cell cultures express relatively lower mRNA levels for ER-α, ER-β, total PR and PR-B. The ER and PR mRNA levels in stromal cells were comparable to those in MCF-7, but higher than malignant endometrial and breast cancer cell lines KLE, AN3, and MDA-MB-231 (Figure 1B). Results of Western blotting analyses confirmed the expression of ER and PR proteins in stromal cells. Relatively high levels of ER-α, ER-β, and low levels of PR-A and PR-B were detected in stromal cells. These results indicated that cultured stromal cells express measurable levels of ER and PR and can therefore be used as a model for studying hormonal regulation.

### 2.2. Morphological Changes Following Hormonal Treatment

**Figure 2:**
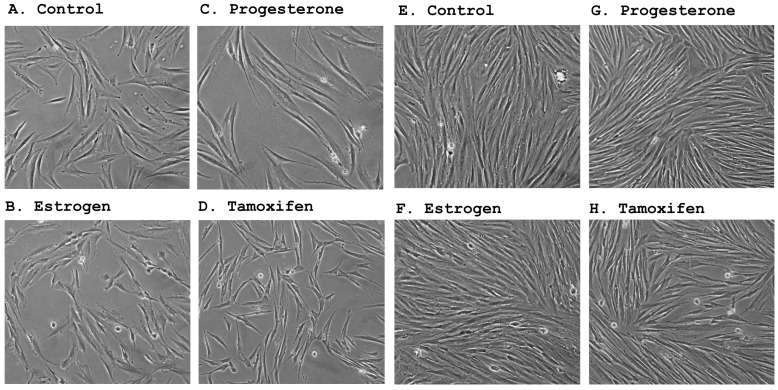
Morphological changes in stromal cells following hormonal treatment. Hormonal effects in low density (Panels (**A**–**D**)) and high density cultures (Panels (**E**–**H**)) were shown; The cells were treated for 48 h with alcohol as solvent control (**A**,**E**); 1 × 10^−7^ M of β-estradiol (**B**,**F**); 2 × 10^−7^ M of progesterone (**C**,**G**); or 2 × 10^−7^ M of tamoxifen (**D**,**H**). Estrogen treated cells became smaller and polymorphic compared to control and progesterone treated cells. Progesterone treated cells were characterized by their larger size and spindle-like shape with smooth edges. Tamoxifen treated cells (panels (**D**) and (**H**)) were relatively small, many in triangular shape. Scale bar = 25 μM.

We observed significant morphological changes in stromal cell cultures following the treatment with ovarian steroids. Estrogen-treated cells became more polymorphic than the control and progesterone treated cells (Figure 2). Clusters of cells with de-differentiated appearance, characterized by a smaller size and round shape, were observed following estrogen treatment. On the other hand, progesterone treated cells became larger with better differentiation. Tamoxifen treated cells became smaller and many cells exhibited a distinctive triangular shape. The divergent morphological changes suggest that these hormones may induce different gene expression patterns in endometrial stromal cells.

### 2.3. Estrogen and Progesterone Induce Different Gene Expression

Using microarray analysis, we compared the gene expression patterns of stromal cell cultures with or without hormonal treatment. Estrogen, progesterone, and tamoxifen up-regulated 788, 1368, and 1027 genes more than 2-fold, respectively. These genes were classified into six categories including structural, enzymes, transcription factors, signaling, cell cycle and apoptosis, and novel genes with unknown functions. Table 1 and Table 2 present a selected list of the genes significantly regulated by estrogen and progesterone (>3-fold), respectively. It is noteworthy that a literature search indicated that only a few of these genes are known for their responses to hormones. A majority of the genes were identified for the first time as hormonal regulation targets.

**Table 1:** Genes responding to estrogen treatment. Representative genes with significant alterations (>3-fold) were compiled into six categories. Gene designation, function, and fold-change are presented. Stromal cells were treated with 1 × 10^−7^ M of β-estradiol for 48 h. Note that decimals indicate down-regulation of gene expression.

Gene Name	Description	E2/Ctl
	**Structural**	
*CNTN4*	contactin 4	0.08
*TRAR1*	trace amine receptor 1	0.10
*COL14A1*	collagen, type XIV, α1 (undulin)	0.14
*SFXN5*	sideroflexin 5	0.15
*PAPLN*	papilin, proteoglycan-like sulfated glycoprotein	0.17
*CDRT1*	CMT1A duplicated region transcript 1	0.29
*SHPRH*	SNF2 histone linker PHD RING helicase	3.10
*OR2W1*	olfactory receptor, family 2, subfamily W, member 1	3.49
*CANP*	cancer-associated nucleoprotein	3.66
*COL9A1*	collagen, type IX, α1	6.06
*HIST2H4*	histone 2, H4	6.28
*SESN3*	sestrin 3	6.34
*TUBB1*	tubulin, β1	9.88
*CLECSF12*	C-type (calcium dependent, carbohydrate-recognition domain) lectin, superfamily member 12	23.48
	**Enzymes**	
*SMA4*	SMA4	0.16
*TLL1*	tolloid-like 1	0.21
*ANG*	angiogenin, ribonuclease, RNase A family, 5	0.22
*B3GALT4*	UDP-Gal:β GlcNAc β 1,3-galactosyltransferase, polypeptide 4	0.24
*PDE11A*	phosphodiesterase 11A	0.25
*ABCG4*	ATP-binding cassette, sub-family G (WHITE), member 4	0.26
*PFKFB2*	6-phosphofructo-2-kinase/fructose-2,6-biphosphatase 2	0.26
*CYP7B1*	cytochrome P450, family 7, subfamily B, polypeptide 1	0.29
*ENPP1*	ectonucleotide pyrophosphatase/phosphodiesterase 1	3.21
*TIGD6*	tigger transposable element derived 6	3.54
*MMRN1*	multimerin 1	5.24
*RTN4IP1*	reticulon 4 interacting protein 1	9.90
*PTPRZ1*	protein tyrosine phosphatase, receptor-type, Z polypeptide 1	14.49
	**Transcription Factors**	
*ZNF608*	zinc finger protein 608	0.13
*TSPAN-2*	tetraspan 2	0.21
*ZNF75*	zinc finger protein 75 (D8C6)	0.24
*ZIC4*	Zic family member 4	0.27
*ZNF423*	zinc finger protein 423	0.31
*POGZ*	pogo transposable element with ZNF domain	0.33
*MXI1*	MAX interactor 1	0.33
*CLDN23*	claudin 23	3.12
*ZNF197*	zinc finger protein 197	3.65
*TTTY15*	testis-specific transcript, Y-linked 15	5.84
	**Signaling**	
*IL12B*	interleukin 12B (natural killer cell stimulatory factor 2, cytotoxic lymphocyte maturation factor 2, p40)	0.07
*STARS*	striated muscle activator of Rho-dependent signaling	0.13
*CFC1*	cripto, FRL-1, cryptic family 1	0.16
*GPR133*	G protein-coupled receptor 133	0.28
*OLFML2A*	olfactomedin-like 2A	0.30
*PTTG2*	pituitary tumor-transforming 2	3.04
*EDG7*	endothelial differentiation, lysophosphatidic acid G-protein-coupled receptor, 7	3.06
*BAGE*	B melanoma antigen	3.08
*MKLN1*	muskelin 1, intracellular mediator containing kelch motifs	3.24
	**Cell cycle and Apoptosis**	
*ID1*	inhibitor of DNA binding 1, dominant negative helix-loop-helix protein	3.82
*CHAF1B*	chromatin assembly factor 1, subunit B (p60)	4.09
*IL8*	interleukin 8	5.18
*ID3*	inhibitor of DNA binding 3, dominant negative helix-loop-helix protein	6.51
	**Novel Genes**	
*FLJ31842*	hypothetical protein FLJ31842	0.06
*FLJ13544*	hypothetical protein FLJ13544	0.18
*LOC338773*	hypothetical protein LOC338773	0.24
*LOC200169*	hypothetical protein LOC200169	7.28
*MGC39830*	hypothetical protein MGC39830	8.20

**Table 2:** Genes responding to progesterone treatment. Representative genes with significant alterations (>3-fold) from different categories were compiled. Gene designation, function, and fold-change are presented. Stromal cells were treated with 2 × 10^−7^ M of progesterone for 48 h.

Gene Name	Description	Prog/Ctl
	**Structural**	
*LMNB1*	lamin B1	0.18
*JUP*	junction plakoglobin	0.18
*SYNPO*	synaptopodin	0.20
*ACTG2*	actin, γ2, smooth muscle, enteric	0.23
*ARHGDIB*	Rho GDP dissociation inhibitor (GDI) β	0.27
*COL11A1*	collagen, type XI, α1	0.28
*ANLN*	anillin, actin binding protein (scraps homolog, Drosophila)	0.32
*ITGA2*	integrin, α2 (CD49B, α2 subunit of VLA-2 receptor)	3.19
*STCH*	stress 70 protein chaperone, microsome-associated, 60 kDa	3.42
*ADFP*	adipose differentiation-related protein	3.71
*TUBE1*	tubulin, epsilon 1	4.44
*NPC1*	Niemann-Pick disease, type C1	5.63
	**Enzymes**	
*SMA4*	SMA4	0.06
*CYP1B1*	cytochrome P450, family 1, subfamily B, polypeptide 1	0.13
*MEST*	mesoderm specific transcript homolog (mouse)	0.13
*PLK4*	polo-like kinase 4 (Drosophila)	0.22
*BRIP1*	BRCA1 interacting protein *C*-terminal helicase 1	0.24
*RNASEL*	ribonuclease L (2',5'-oligoisoadenylate synthetase-dependent)	0.31
*ASNS*	asparagine synthetase	3.01
*NNMT*	nicotinamide *N*-methyltransferase	3.43
*HS3ST3A1*	heparan sulfate (glucosamine) 3-*O*-sulfotransferase 3A1	3.72
*LIAS*	lipoic acid synthetase	7.07
*PRKY*	protein kinase, Y-linked	9.49
*JAK2*	Janus kinase 2 (a protein tyrosine kinase)	10.82
	**Transcription Factors**	
*SOX4*	SRY (sex determining region Y)-box 4	0.15
*ZNF323*	zinc finger protein 323	0.17
*FOXL2*	forkhead box L2	0.33
*TBX2*	T-box 2	3.01
*E2F7*	E2F transcription factor 7	3.04
*DDIT4*	DNA-damage-inducible transcript 4	3.31
*TGIF*	TGFB-induced factor (TALE family homeobox)	3.44
*FOXD1*	forkhead box D1	3.78
*ZNF197*	zinc finger protein 197	3.84
*DSCR1*	Down syndrome critical region gene 1	3.88
*ID3*	inhibitor of DNA binding 3, dominant negative helix-loop-helix protein	5.25
*DDIT3*	DNA-damage-inducible transcript 3	8.18
*TCF21*	transcription factor 21	13.97
	**Signaling**	
*TACSTD1*	tumor-associated calcium signal transducer 1	0.21
*PIR51*	RAD51 associated protein 1	0.26
*FGF9*	fibroblast growth factor 9 (glia-activating factor)	0.29
*RHOB*	ras homolog gene family, member B	0.31
*IL6*	interleukin 6 (interferon, β2)	3.28
*VEGF*	vascular endothelial growth factor	3.39
*HSPBAP1*	HSPB (heat shock 27 kDa) associated protein 1	3.47
*AREG*	amphiregulin (schwannoma-derived growth factor)	3.67
*GDF15*	growth differentiation factor 15	4.49
*GADD45A*	growth arrest and DNA-damage-inducible, α	5.02
*RIS1*	Ras-induced senescence 1	6.32
	**Cell cycle and Apoptosis**	
*CDC25A*	cell division cycle 25A	0.13
*SKP2*	S-phase kinase-associated protein 2 (p45)	0.24
*CCNE2*	cyclin E2	0.29
*CDKN2B*	cyclin-dependent kinase inhibitor 2B (p15, inhibits CDK4)	3.12
*SH3MD2*	SH3 multiple domains 2	3.29
*BTG1*	B-cell translocation gene 1, anti-proliferative	3.50
	**Novel Genes**	
*FLJ11795*	hypothetical protein FLJ11795	0.04
*LOC283112*	hypothetical protein LOC283112	0.05
*LOC145741*	hypothetical LOC145741	7.70
*FLJ11011*	hypothetical protein FLJ11011	7.95
*MGC39830*	hypothetical protein MGC39830	8.94

Further data analysis focused on comparing the expression patterns induced by different hormones. Estrogen and progesterone treatment caused dramatic, but different alterations in gene expression. Among the 2156 genes with more than 2-fold expression alterations, 325 of them (15.1%) were regulated by both hormones; furthermore, one third (109, 33.5%) of the genes from this group were regulated differentially by the two hormones, with regulation by one hormone for more than 2-fold, but not by another. Table 3 lists some of the genes whose mRNA levels were differentially affected by estrogen and progesterone. Interestingly, 16 genes were found to be regulated in opposite directions by β-estradiol and progesterone, that is, up-regulated by one hormone for more than 2-fold, and down-regulated by another for more than 2-fold. As discussed later, these genes may be part of the regulatory network responsible for the opposing effects of estrogen and progesterone in the uterus.

In contrast to the divergent effects of estrogen and progesterone, the expression pattern induced by estrogen and tamoxifen appeared to be more similar. 23.4% (425 out of 1815) of genes were common targets for both hormones. Further analysis indicated that more than half (214 out of the 425) of these common genes were regulated in the same direction by estrogen and tamoxifen for more than 2-fold. Representative genes are listed in Table 4. Despite their similarity, the expression patterns induced by tamoxifen and estrogen were not identical. Table 5 listed representative genes with differential responses to the two hormones. Thus, tamoxifen displays similar, but distinct effects on stromal cells compared to estrogen.

**Table 3:** Genes differentially regulated by estrogen and progesterone. Genes regulated in opposite directions by the two hormones (more than 2-fold) were marked with asterisks. The rest of the genes were significantly regulated by one, but not another hormone. The cells were treated for 48 h with either 1 × 10^−7^ M of β-estradiol or 2 × 10^−7^ M of progesterone.

Gene Name	Description	E2/Ctl	Prog/Ctl
	**Structural**		
*COL4A3BP*	collagen, type IV, α3 (Goodpasture antigen) binding protein	0.62	2.05
*MYLIP*	myosin regulatory light chain interacting protein	0.63	2.56
*LAMA1*	laminin, α1	0.69	2.17
*TUBE1*	tubulin, epsilon 1	0.71	4.44
*HIST1H2AC*	histone 1, H2ac	0.84	2.71
*CHS1*	Chediak-Higashi syndrome 1	1.09	2.14
*H2AFX*	H2A histone family, member X	1.69	0.47
*KIF20A*	kinesin family member 20A	2.23	0.44
*TMPO*	thymopoietin	2.24	0.61
*OIP5*	Opa-interacting protein 5	2.26	0.52
*HCAP-G*	chromosome condensation protein G	2.41	0.65
*NEK2*	NIMA (never in mitosis gene a)-related kinase 2	2.45	0.58
MAD2L1 *	MAD2 mitotic arrest deficient-like 1 (yeast)	2.56	0.47
	**Enzymes**		
*SCD*	stearoyl-CoA desaturase (δ-9-desaturase)	0.43	1.96
*HMGCR*	3-hydroxy-3-methylglutaryl-Coenzyme A reductase	0.44	1.97
*ASNS*	asparagine synthetase	0.66	3.01
*EIF2AK3*	eukaryotic translation initiation factor 2-α kinase 3	0.81	2.58
*PRIM2A*	primase, polypeptide 2A, 58 kDa	1.54	0.47
*FEN1*	flap structure-specific endonuclease 1	1.93	0.44
*POLA2*	polymerase (DNA-directed), α (70 kD)	2.17	0.84
TOP2A *	topoisomerase (DNA) II α 170 kDa	2.26	0.41
DNA2L *	DNA2 DNA replication helicase 2-like (yeast)	2.43	0.37
POLE2 *	polymerase (DNA directed), epsilon 2 (p59 subunit)	2.52	0.48
PLK4 *	polo-like kinase 4 (Drosophila)	2.56	0.22
*TOPK*	T-LAK cell-originated protein kinase	2.77	0.51
	**Transcription Factors**		
DDIT4 *	DNA-damage-inducible transcript 4	0.35	3.31
*DDIT4L*	DNA-damage-inducible transcript 4-like	0.69	2.01
*ATF4*	activating transcription factor 4 (tax-responsive enhancer element B67)	0.69	2.26
*DSCR3*	Down syndrome critical region gene 3	0.82	2.10
*ATF3*	activating transcription factor 3	0.85	3.65
*MAFF*	v-maf musculoaponeurotic fibrosarcoma oncogene homolog F (avian)	0.87	2.45
*NFIL3*	nuclear factor, interleukin 3 regulated	0.88	3.74
*TGIF*	TGFB-induced factor (TALE family homeobox)	0.90	3.44
*OLIG1*	oligodendrocyte transcription factor 1	2.16	0.85
*TCF19*	transcription factor 19 (SC1)	2.43	0.83
*PTTG1*	pituitary tumor-transforming 1	2.48	0.67
*FOXM1*	forkhead box M1	2.53	0.82
*MYBL1*	v-myb myeloblastosis viral oncogene homolog (avian)-like 1	2.60	0.66
	**Signaling**		
STARS *	striated muscle activator of Rho-dependent signaling	0.13	2.25
RAB33A *	RAB33A, member RAS oncogene family	0.42	3.00
*NGFB*	nerve growth factor, β polypeptide	0.58	2.69
*GDF15*	growth differentiation factor 15	0.67	4.49
*MAP4K3*	mitogen-activated protein kinase kinase kinase kinase 3	0.75	2.04
*VEGF*	vascular endothelial growth factor	0.85	3.39
*MTSS1*	metastasis suppressor 1	0.90	3.72
*GADD45A*	growth arrest and DNA-damage-inducible, α	0.92	5.02
*DNAJB9*	DnaJ (Hsp40) homolog, subfamily B, member 9	0.95	6.25
*SHCBP1*	SHC SH2-domain binding protein 1	2.05	0.41
*MYCBP*	c-myc binding protein	2.06	0.73
*ECT2*	epithelial cell transforming sequence 2 oncogene	2.35	0.65
*PIR51*	RAD51 associated protein 1	2.45	0.26
	**Cell cycle and Apoptosis**		
*KLF4*	Kruppel-like factor 4 (gut)	0.51	2.93
*CDKN2B*	cyclin-dependent kinase inhibitor 2B (p15, inhibits CDK4)	0.52	2.92
CCNE2 *	cyclin E2	2.00	0.29
*FANCG*	Fanconi anemia, complementation group G	2.14	0.70
*CDC2*	cell division cycle 2, G1 to S and G2 to M	2.15	0.53
CDCA2 *	cell division cycle associated 2	2.17	0.48
MTB *	more than blood homolog	2.23	0.49
*CCNA2*	cyclin A2	2.31	0.53
CCNB1 *	cyclin B1	2.32	0.48
CDCA3 *	cell division cycle associated 3	2.34	0.45
*CDKN3*	cyclin-dependent kinase inhibitor 3 (CDK2-associated dual specificity phosphatase)	2.40	0.69
*CCNB2*	cyclin B2	2.45	0.55
*CDC20*	CDC20 cell division cycle 20 homolog (*S. cerevisiae*)	3.29	0.58
	**Novel Genes**		
FLJ20366 *	hypothetical protein FLJ20366	0.38	2.73
LOC153346 *	hypothetical protein LOC153346	0.46	2.71
FLJ20105 *	hypothetical protein FLJ20105	2.77	0.33
*FLJ10719*	hypothetical protein FLJ10719	3.22	0.52
*FLJ13273*	hypothetical protein FLJ13273	4.82	0.67

**Table 4:** Genes responded in a similar way to estrogen and tamoxifen treatment. Information on representative genes that were up- or down-regulated by both estrogen and tamoxifen were listed. The cells were either treated for 48 h with 1 × 10^−7^ M of β-estradiol or 2 × 10^−7^ M of tamoxifen.

Gene Name	Description	E2/Ctl	Tam/Ctl
	**Structural**		
*CRABP2*	cellular retinoic acid binding protein 2	0.33	0.49
*KCTD7*	potassium channel tetramerisation domain containing 7	0.38	0.39
*FLRT2*	fibronectin leucine rich transmembrane protein 2	0.39	0.47
*DAAM2*	dishevelled associated activator of morphogenesis 2	0.46	0.48
*CHAF1A*	chromatin assembly factor 1, subunit A (p150)	2.00	2.44
*CNAP1*	chromosome condensation-related SMC-associated protein 1	2.01	2.45
*KNTC2*	kinetochore associated 2	2.02	2.92
*ITGA2*	integrin, α2 (CD49B, α2 subunit of VLA-2 receptor)	2.16	2.86
*OIP5*	Opa-interacting protein 5	2.26	2.47
*RAMP*	RA-regulated nuclear matrix-associated protein	2.40	3.09
*HCAP-G*	chromosome condensation protein G	2.41	2.96
*NEK2*	NIMA (never in mitosis gene a)-related kinase 2	2.45	2.46
*MAD2L1*	MAD2 mitotic arrest deficient-like 1 (yeast)	2.56	2.22
*PODXL*	podocalyxin-like	2.69	3.95
	**Enzymes**		
*SMA4*	SMA4	0.16	0.42
*MMP11*	matrix metalloproteinase 11 (stromelysin 3)	0.48	0.41
*RRM2*	ribonucleotide reductase M2 polypeptide	2.09	2.35
*RNASEH2A*	ribonuclease H2, large subunit	2.12	2.60
*POLA2*	polymerase (DNA-directed), α (70 kD)	2.17	3.08
*TOP2A*	topoisomerase (DNA) II α 170 kDa	2.31	2.60
*POLE2*	polymerase (DNA directed), epsilon 2 (p59 subunit)	2.52	3.01
*CDKN3*	cyclin-dependent kinase inhibitor 3 (CDK2-associated dual specificity phosphatase)	2.62	2.64
*HAS2*	hyaluronan synthase 2	2.72	4.27
*MMP1*	matrix metalloproteinase 1 (interstitial collagenase)	2.73	8.91
*PLK1*	polo-like kinase 1 (Drosophila)	2.75	3.86
*BUB1*	BUB1 budding uninhibited by benzimidazoles 1 homolog (yeast)	2.81	2.48
	**Transcription Factors**		
*POGZ*	pogo transposable element with ZNF domain	0.33	0.39
*KLF4*	Kruppel-like factor 4 (gut)	0.42	0.48
*PBXIP1*	pre-B-cell leukemia transcription factor interacting protein 1	0.44	0.49
*RFC4*	replication factor C (activator 1) 4, 37 kDa	2.03	2.69
*TCF19*	transcription factor 19 (SC1)	2.43	2.16
*PTTG1*	pituitary tumor-transforming 1	2.48	2.95
*FOXM1*	forkhead box M1	2.53	3.39
*MYBL1*	v-myb myeloblastosis viral oncogene homolog (avian)-like 1	2.60	2.60
*ID1*	inhibitor of DNA binding 1, dominant negative helix-loop-helix protein	3.82	4.37
	**Signaling**		
*DDIT4*	DNA-damage-inducible transcript 4	0.35	0.26
*RASSF2*	Ras association (RalGDS/AF-6) domain family 2	0.44	0.38
*RASGRP1*	RAS guanyl releasing protein 1 (calcium and DAG-regulated)	0.50	0.37
*FBXO5*	F-box protein 5	2.20	2.16
*RANBP1*	RAN binding protein 1	2.21	2.11
*ECT2*	epithelial cell transforming sequence 2 oncogene	2.35	2.09
*PIR51*	RAD51 associated protein 1	2.45	2.34
*RGS4*	regulator of G-protein signalling 4	2.94	3.81
*IL8*	interleukin 8	5.18	2.00
	**Cell cycle and Apoptosis**		
*TRIB3*	tribbles homolog 3 (Drosophila)	0.47	0.35
*GTSE1*	G-2 and S-phase expressed 1	2.07	2.42
*FANCG*	Fanconi anemia, complementation group G	2.14	2.23
*CDC2*	cell division cycle 2, G1 to S and G2 to M	2.20	2.12
*MTB*	more than blood homolog	2.23	2.20
*CCNA2*	cyclin A2	2.31	2.70
*CCNB1*	cyclin B1	2.32	2.71
*CCNB2*	cyclin B2	2.45	2.83
*CDCA1*	cell division cycle associated 1	2.61	3.56
*FANCE*	Fanconi anemia, complementation group E	2.95	2.62
	**Novel Genes**		
*LOC338773*	hypothetical protein LOC338773	0.24	0.43
*KIAA1164*	hypothetical protein KIAA1164	0.31	0.34
*DKFZP434L142*	hypothetical protein DKFZp434L142	0.39	0.34
*FLJ10719*	hypothetical protein FLJ10719	3.22	4.02
*FLJ13273*	hypothetical protein FLJ13273	4.82	3.96

**Table 5:** Genes differentially regulated by estrogen and tamoxifen. Representative genes that were differentially regulated by estrogen and tamoxifen were listed. The cells were treated for 48 h with 1 × 10^−7^ M of β-estradiol or 2 × 10^−7^ M of tamoxifen.

Gene Name	Description	E2/Ctl	Tam/Ctl
	**Structural**		
*ARRDC2*	arrestin domain containing 2	0.58	2.34
*SLC16A6*	solute carrier family 16 (monocarboxylic acid transporters), member 6	0.77	2.33
*SYCP2*	synaptonemal complex protein 2	2.48	0.84
	**Enzymes**		
*PIN1*	protein (peptidyl-prolyl cis/trans isomerase) NIMA-interacting 1	0.72	2.21
	**Transcription Factors**		
*OLIG1*	oligodendrocyte transcription factor 1	2.16	0.86
	**Signaling**		
*NOV*	nephroblastoma overexpressed gene	0.83	2.00
	**Novel Genes**		
*FLJ38993*	hypothetical protein FLJ38993	0.77	2.01

### 2.4. Validation of Microarray Results

**Figure 3:**
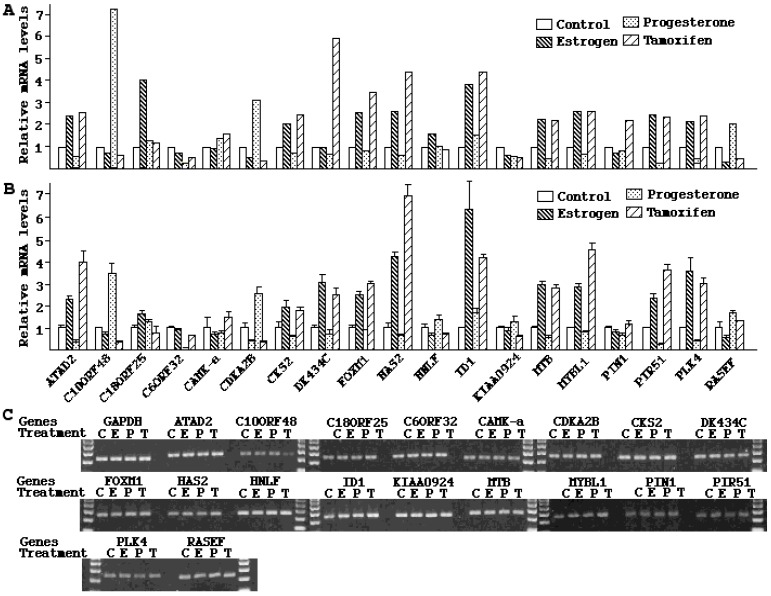
Validation of microarray results. 19 genes were chosen as example genes for confirmation by real-time PCR. For each gene, relative mRNA levels in control, estrogen, progesterone and tamoxifen groups measured by microarray (**A**) and real-time PCR (**B**) were shown. The real-time PCR results represented average values from four parallel reactions; (**C**) Agarose gel electrophoresis of the end products (40 cycles) from real-time PCR. The single band patterns with predicted sizes indicated the high specificity of PCR.

To verify the reliability of the microarray results, 19 sample genes were selected for real-time PCR analysis. These genes were chosen by their novelty and significant responses to different hormones. We replicated the same experimental conditions as those applied for microarray analysis, and measured mRNA levels of these genes. Each real-time PCR result was standardized by the correspondent *GAPDH* mRNA level (Figure 3B) and compared to the microarray results (Figure 3A). High PCR specificity was confirmed by agarose gel electrophoresis of the amplification products from the 40th cycle of real-time PCR (Figure 3C). The real-time PCR and microarray results were consistent in most of the genes tested, providing strong support for the overall reliability of the microarray data.

## 3. Discussion

The expression of estrogen and progesterone receptors in human endometrial stromal cells has been reported by several groups [22,23,24,25]. These previous observations, however, were mostly based on immunohistochemistry studies [24,25], and the receptors’ expression levels relative to other tissues were not compared. In this study, we performed real-time PCR and Western blotting analysis to compare mRNA and protein levels between endometrial stromal cells and several reproductive tissues. In general, stromal cells appeared to express substantial amounts of estrogen and progesterone receptors, a result consistent with most previous reports [22].

In accordance with their estrogen and progesterone receptor expression status, primary stromal cultures underwent significant morphological changes following treatment by steroid hormones. The counteraction of estrogen and progesterone appeared to be reminiscent of their opposing functions observed during the menstrual cycle where estrogen is responsible for cell proliferation [26,27] and progesterone for cell differentiation [28,29,30]. Accompanying the morphological changes were dramatic and divergent alterations in mRNA expression by the two hormones. The opposite effects of estrogen and progesterone are best illustrated by the results from a number of cyclins. As shown in Table 4 under cell cycle and apoptosis genes, *cyclin A2*, *B1* and *B2* were all up-regulated by estrogen for more than 2-fold, but down-regulated by progesterone. These mitogens are known to promote cell proliferation and stimulate malignant transformation. *Cyclin A2* activation is considered a prerequisite for G1/S and G2/M transition [31]. Elevated expression of *cyclin A2* and their catalytic partners *CDK1* and *CDK2* have been detected in male germ cell tumors, and their levels are quantitatively associated with tumor invasiveness [32]. Over-expression of a non-degradable form of *cyclin A2* (δ 152) affected the cell cycle and promoted aneuploidy as well as transformation of rat fibroblasts [33]. On the other hand, decreased levels of *cyclin A2* and *B1* were associated with P53-dependent G2 arrest in human squamous carcinoma cells [34]. Therefore, estrogen-induced up-regulation of cyclins may be partially responsible for the carcinogenic effects observed on human endometrium and stroma. Their down-regulation by progesterone may contribute to this hormone’s cytostatic effect in the uterine cells. Since both stromal sarcomas and endometrial cancers respond to progestational treatment [35,36,37], further exploration on the regulation and function of these genes may potentially lead to the improvement of hormonal prevention and/or treatment of uterine malignancies.

DNA synthesis represents a critical step in cell proliferation. A series of enzymes required for DNA replication such as DNA helicase, topoisomerase, and polymerase, were significantly affected by estrogen and progesterone. The DNA helicase complex functions as a replication licensing system that ensures precise chromosomal DNA replication before cell division [38]. DNA topoisomerase II is required for chromosome segregation following DNA replication [39]. DNA polymerase α and epsilon are the two major eukaryotic processing polymerases that are recruited to replication origins during the late G1 phase [40]. Drug inhibition of DNA polymerase α and epsilon led to suppression of cancer cell growth [41]. Lee *et al.* reported that a human trophoblast cell line transfected with SV40 T antigen displayed increased proliferation and invasiveness, but was unable to form colonies on soft agar or tumors in nude mice. Interestingly, in these premalignant cells, DNA polymerase epsilon was identified as one of the genes up-regulated compared to the untransfected control. In this study, DNA helicase-2, DNA polymerase α and epsilon, and DNA topoisomerase II were all up-regulated by estrogen, but down-regulated by progesterone (Table 3, Enzymes), suggesting that these enzymes may play important role(s) in the estrogen-dependent neoplastic diseases of the uterus [42].

The estrogenic and carcinogenic effects of tamoxifen in stromal and endometrial cells have been well documented [15,27]. Since tamoxifen has been used to prevent breast cancer recurrence in high-risk women, investigating its effects on stromal cells bears strong clinical significance. Characterization of gene expression signatures represents the first step to better understand tamoxifen-mediated tumorigenic effects in the endometrium. Our data indicate that gene expression patterns induced by tamoxifen share some similarity with those of estrogen. In fact, all the genes discussed above, including those of *cyclin A2*, *B1* and *B2*, DNA polymerase α and epsilon, and DNA topoisomerase II were found to be up-regulated by both estrogen and tamoxifen (Table 4, enzymes, cell cycle and apoptosis genes). It was reported that the subtle structural differences of estrogen and tamoxifen cause distinct conformational changes in the ER upon binding by these hormones [43,44], leading to the recruitment of alternative co-activators into the transcription initiation complex [45,46]. The few genes differentially regulated by estrogen and tamoxifen may provide an ideal model to study the distinct transactivation mechanisms by estrogen and tamoxifen.

Estrogen and progesterone concentrations undergo dynamic changes under different physio-pathological conditions. In addition, stromal cells were exposed simultaneously to both estrogen and progesterone, the combined effects by the two hormones added another dimension to the complexity of hormonal regulation. In this study, one hormone a time at a single concentration was examined for each hormone. In addition, stromal-epithelial interactions were not considered when purified stromal cells were used. Results from experiments applying these much-simplified conditions could not accurately reflect the *in vivo* situation. Thus, the observations needs to be further verified under physiological conditions. Nevertheless, the microarray analysis led to the identification of numerous candidate target genes, which provided useful information for future mechanistic and functional studies concerning the hormonal regulation in the endometrial stromal cell population.

## 4. Material and Methods

### 4.1. Tissue, Cell Lines, and Reagents

Normal breast, ovarian and endometrium tissues were collected from patients with benign conditions. Endometrial stromal cells were isolated by collagenase/DNase I digestion following established protocols [47,48]. Endometrial adenocarcinoma cell lines KLE and AN3, and breast cancer cell lines MCF-7 and MDA-MB-231 were obtained from American Type Culture Collection (Rockville, MD, USA). Stromal KLE and cells were grown in F12 medium. AN3, MCF-7, and MDA-MB-231 cells were maintained in MEM medium. All the media were supplemented with 10% FCS (BioWhittaker, Walkersville, MD, USA), 100 µg/mL streptomycin, 100 units/mL penicillin, and 2 mM glutamine. Cell cultures were maintained at 37 °C in an atmosphere containing 5% CO_2_ and 100% humidity.

β-estradiol, progesterone, tamoxifen, and monoclonal antibody for ER-β (SAB4500814) and goat anti-rabbit secondary antibody (A4062) were purchased from Sigma (St. Louis, MO, USA). Antibodies against ER-α (MC-20), PR-A or PR-B (C-20) were products of Santa Cruz Biotechnology (Santa Cruz, CA, USA). Rabbit anti-β-actin antibody (TA306308) was purchased from Oncogene (Boston, MA, USA).

### 4.2. Cell Treatment, RNA Isolation, and Quantitative Real-Time PCR

Stromal cell cultures were grown in 10 cm diameter dishes. Cells were de-induced in medium containing 10% charcoal-stripped serum for two days before treated with 1 × 10^−7^ M of β-estradiol, 2 × 10^−7^ M of tamoxifen, or 2 × 10^−7^ M of progesterone for 48 h. Total RNA was isolated using Trizol reagents (Invitrogen, Carlsbad, CA, USA). The RNA samples were treated with DNA Free™ (Ambion, Austib, TX, USA) to eliminate contaminated genomic DNA. cDNA was synthesized from 1 µg of RNA with random primers using SuperScript kit (Invitrogen, Carlsbad, CA, USA). The 20 µL of reverse transcription products were diluted to 100 and 2 µL was used for each real-time PCR. PCR reactions were carried out in 25 µL containing 140 ng of primers and 12.5 µL SYBR Green Master Mix (Stratagene, Cedar Creek, TX, USA). The designations and sequences of PCR primers are described in Table 6. Real-time PCR was performed under the following conditions: initial denature, 95 °C for 10 min, followed by 40 cycles of denaturation at 95 °C for 30 s, annealing at 56 °C for 40 s, and extension at 72 °C for 30 s. The threshold cycle number (*C*_t_) values were standardized against *GAPDH* controls, converted to fold (2*^C^*^t^) relative to *GAPDH*, and compared between experimental and control groups. All data groups were analyzed by ANOVA to determine if there was significance (*p* < 0.05) among the groups. For all experimental groups that satisfied the initial ANOVA criterion, individual comparisons were performed with the use of *post hoc* Bonferroni *t* tests based on assumptions of two-tailed distribution and two samples with equal variance. Statistical significance (*p* ≤ 0.05) is indicated by asterisks in the figures.

**Table 6:** Primers and sizes of amplicons in real-time PCR.

Gene Name	Description	5' Primer	3' Primer	Size (bp)
*ER-α*	Estrogen receptor α	aattcagataatcgacgccag	gtgtttcaacattctccctcctc	344
*ER-β*	Estrogen receptor β	tgcggaacctcaaaagagtc	cttcacacgaccagactcca	206
*PR-AB*	Progesterone receptor A and B	atgagccggtccgggtgcaag	gccacccagagcccgaggttt	243
*PR-B*	Progesterone receptor B	gactgagagcttcacagtat	tctcctaactcggggagttct	187
*ATAD2*	ATPase family, AAA domain containing 2	gattatcttccgcaggacca	gttgcattggatcaacatcg	255
*C10ORF48*	chromosome 10 open reading frame 48	gggtcaatagtgcagccagt	tgcgcttactgttactgcaaa	247
*C18ORF25*	chromosome 18 open reading frame 25	gtaggggccagactgaatga	agtgtccccagctttttcaa	250
*C6ORF32*	chromosome 6 open reading frame 32	aggagaaaatgccactgtcg	tcctctgggtcttcctcctt	250
*CAMK-a*	calcium/calmodulin-dependent protein kinase II	acgagaagctgagcccctac	ttgggggagttagacaccag	221
*CDKN2B*	cyclin-dependent kinase inhibitor 2B (p15, inhibits CDK4)	tcgtttgcttttcagggttt	cctcctccactttgtcctca	248
*CKS2*	CDC28 protein kinase regulatory subunit 2	ggagtggaggagacttggtg	cagctcatgcacaggtatgg	236
*DK434C*	DKFZP434C245 protein	taagctgtgggacaagagca	ttgagtcactggaggctgtg	248
*FOXM1*	forkhead box M1	cgtggattgaggaccacttt	gattcggtcgtttctgctgt	249
*HAS2*	hyaluronan synthase 2	agagcactgggacgaagtgt	atgcactgaacacacccaaa	245
*HNLF*	putative NFkB activating protein HNLF	agaagcgctgtttcatcgag	gccatcctggtagaattgga	253
*ID1*	inhibitor of DNA binding 1, dominant negative helix-loop-helix protein	cccattctgtttcagccagt	agccgttcatgtcgtagagc	245
*KIAA0924*	KIAA0924 protein	atcgctcattttgaggttgc	gcagaggacagggcagtaaa	246
*MTB*	more than blood homolog	tgcgggaggttctgagttac	ggaccatcgggtaaggatct	261
*MYBL1*	v-myb myeloblastosis viral oncogene homolog (avian)-like 1	gtccgaaacgttggtctgtt	gaccttccgacgcattgtag	248
*PIN1*	protein (peptidyl-prolyl cis/trans isomerase) NIMA-interacting 1	tgccaccgtcacacagtatt	gagtctgcctccagcacct	253
*PIR51*	RAD51 associated protein 1	ttctggaaggcagtgatggt	gagcagagtccaccgaagtc	243
*PLK4*	polo-like kinase 4 (Drosophila)	gccaaggaccttattcacca	ttatttgggagtggctgacc	251
*RASEF*	RAS and EF hand domain containing	atcaaccttgtggagccaag	ctgaggtcactgagggcttc	245

### 4.3. Western Blot Analysis

Cell extracts (20 µg) were resolved in SDS polyacrylamide gels (Ready Gel, 4%–15% gradient, Bio-Rad Laboratories, Hercules, CA, USA) and electrically transferred onto an Immun-Blot polyvinylidene difluoride membrane (Bio-Rad Laboratories, Hercules, CA, USA). The membranes were blocked for 2 h in PBS buffer containing 0.1% Tween-20 and 10% nonfat dried milk. Specific antibodies against ER-α, ER-β, PR-A and PR-B, or β-actin were applied following the manufacturer’s recommendations. Primary antibody binding was performed at 4 °C overnight with constant rotation. The secondary antibody binding was carried out at room temperature for 1 h at 1:5000 dilutions. Immunobloting signals were detected using the Chemiluminescence Plus Western Blotting Detection System (Amersham Corp., Arlington Heights, IL, USA). The blots were re-probed with β-actin antibody and the results provided controls for protein loading.

### 4.4. Microarray Hybridization

Affymetrix GeneChip™ Human Genome U133 Plus 2.0 microarrays were used for mRNA profiling. Microarray analysis was performed at Mayo Microarray Core facilities by technologists following standard procedures. Briefly, RNA samples were subject to Agilent analysis for quality controls. cDNA was prepared from 10 μg of RNA, quantified by spectrometry, and used as a template for the synthesis of biotinylated cRNA using RNA transcript labeling reagent (Affymetrix, Santa Clara, CA, USA). The quality of the cRNA probes was verified by gel electrophoresis and pilot hybridization with the Test-3 array. Hybridization solution containing fragmented cRNA probes and control cRNA (BioB, BioC, and BioD) was supplemented with herring sperm DNA and bovine serum albumin. The probe solution was heated at 99 °C for 5 min followed by incubation at 45 °C for 5 min before use. Hybridization was carried out at 45 °C for 16 h with constant rotation at 60 rpm. The arrays were washed and stained with streptavidin-phycoerythrin (Molecular Probes, Eugene, OR, USA). After washes, arrays were scanned using the GeneChip system confocal scanner (Hewlett Packard, Palo Alto, CA, USA).

### 4.5. Microarray Data Analysis

Gene expression profiles were analyzed at the Mayo General Clinical Research Center Genomics, Proteomics, and Metabolic Core Facility using established protocols [49,50]. The GeneChip 5.0 (Affymetrix) program was used to scan and quantitatively document the hybridization signals. Compilation of candidate genes and calculation of changes were performed on SpotFire and Microsoft Excel programs. To minimize the false-positive conclusion, only genes with hybridization signal reached an absolute level that was significantly higher than that of the background (*p* < 0.05) entered analyses. Ingenuity Pathway Analysis (Ingenuity Systems), and the Entrez Search Engine website, from the NCBI (http://www.ncbi.nlm.nih.gov/) were applied to selected genes for analysis on their biological interactions.
